# The origin and distribution of ‘Kokubu’-type splice-site mutations of the *MLO* genes in tobacco varieties

**DOI:** 10.1270/jsbbs.22001

**Published:** 2022-07-01

**Authors:** Masao Arai, Tomoyuki Komatsu, Hisashi Udagawa, Tomoyuki Tajima, Seiki Sato

**Affiliations:** 1 Leaf Tobacco Research Center, Japan Tobacco Inc., 1900 Idei, Oyama, Tochigi 323-0808, Japan

**Keywords:** cultivar ‘Kokubu’, Japanese domestic tobacco, leaf shape, *MILDEW LOCUS O*, powdery mildew resistance, splice-site mutations

## Abstract

The Japanese domestic tobacco (*Nicotiana tabacum* L.) cultivar ‘Kokubu’ shows high powdery mildew resistance that is controlled by splice-site mutations of two *MILDEW LOCUS O* genes, *NtMLO1* and *NtMLO2*. We investigated the existence of the same *NtMLO1/2* splice mutations in the genomes of various tobacco varieties cultivated in Japan and other countries. In total, 14 Japanese domestic cultivars, which were mainly distributed in Kagoshima, had splice-site mutations in both *NtMLO1* and *NtMLO2*. In addition, tobacco cultivars containing only the *NtMLO1* splice-site mutation were found in various tobacco production areas in Japan, but no cultivars with only the *NtMLO2* splice-site mutation were detected. Moreover, the *NtMLO1* splice-site mutation was detected in native Asian, Oriental and cigar tobacco varieties. Consequently, we speculate that these powdery mildew-resistant tobacco cultivars were generated relative recently in the Kagoshima area when a spontaneous mutation occurred at the *NtMLO2* splice site in a cultivar already containing the *NtMLO1* splice-site mutation and that the *NtMLO1* splice-site mutation occurred during the early period of tobacco seed dissemination from the Americas to Asia and Japan.

## Introduction

Tobacco (*Nicotiana tabacum* L.) is a Solanaceae plant that originated from tropical or subtropical America but is now commercially cultivated in more than 120 countries. It is thought that tobacco seeds were initially introduced into Japan by the Portuguese or Spaniards in the 16^th^–17^th^ centuries, but this has not been confirmed. Tobacco plant cultivation and smoking tobacco quickly spread in Japan (Handa 2009). The Edo Shogunate of Japan banned or restricted tobacco cultivation several times to secure annual tributes of rice. These policies led to the cultivation of tobacco in isolated mountains and remote areas that were not fit for rice farming, which in turn led to the development and accumulation of technologies unique to each habitat. The plant type and leaf shape became differentiated by the selection of tobacco suitable for local weather and climatic conditions. Additionally, the taste and aroma were diversified by the development of drying methods, and so-called domestic cultivars were gradually established in each region (Ohashi 1984). According to a survey by the Leaf Tobacco Division, Ministry of Finance conducted in 1898, there were more than 170 domestic tobacco cultivars in Japan, and these cultivars have been divided into six groups (‘Daruma’, ‘Nakano’, ‘Usuha’, ‘Hatano’, ‘Suifu’ and ‘Kokubu’) on the basis of leaf shape (Ohashi 1984).

The Japanese domestic tobacco cultivar ‘Kokubu’ was isolated as a new variety from the fields of the Kokubu area of Kagoshima (Satsuma) during the Bunka Period (1804–1818) (Eyama 1992). Kagoshima is a famous tobacco cultivation area of Japan, and several other domestic cultivars, such as ‘Maru’, ‘Izumi’, ‘Tarumizu’ and ‘Ibusuki’, were cultivated in the past. Because the cured leaves of cultivar ‘Kokubu’ have superior characteristics for *kiseru* smoking, Kokubu became a famous tobacco production area of Japan.

The cultivar ‘Kokubu’ is resistant to the plant fungal disease powdery mildew owing to recessive alleles at two loci. This resistance was identified by Wan (1962), and Fujimura *et al.* (2016) revealed that this resistance results from splice-site mutations of two *MILDEW LOCUS O* genes, *NtMLO1* and *NtMLO2*. The *MLO* genes encode a plant-specific seven-transmembrane domain protein that localizes to the plasma membrane (Devoto *et al.* 2003). The *N. tabacum* genome contains 15 *MLO* genes forming seven clades, and the two *MLO* genes of clade V, *NtMLO1* and *NtMLO2*, are related to powdery mildew susceptibility (Appiano *et al.* 2015). Splice-site mutations in the *NtMLO1* and *NtMLO2* genes of cultivar ‘Kokubu’ confer resistance to powdery mildew owing to the resulting deletions or insertions in the transcripts of both genes that lead to the production of incorrect transcripts (Fujimura *et al.* 2016). Powdery mildew resistance as a result of loss-of-function *MLO* alleles has been observed in various dicots and monocots, such as pea (Pavan *et al.* 2011), *Arabidopsis* (Consonni *et al.* 2006), tomato (Bai *et al.* 2008), cucumber (Nie *et al.* 2015) and barley (Büschges *et al.* 1997). The powdery mildew resistance trait of ‘Kokubu’ has been introduced into the various modern tobacco varieties (Hamamura *et al.* 1981).

Furthermore, some other domestic tobacco cultivars of Japan are powdery mildew resistant (Ohashi 1984), and many, such as ‘Maru’ and ‘Izumi’, originated in Kagoshima. Thus, we hypothesized that the powdery mildew resistant loci of such domestic cultivars may have been introduced from ‘Kokubu’. Because this resistance is caused by the two natural recessive mutations in the *NtMLO1/2* genes, it is reasonable to assume that ‘Kokubu’ was either generated by crossing two varieties each having a splice-site mutation in either the *NtMLO1* or *NtMLO2* gene, or by a spontaneous mutation of the *NtMLO* gene in a variety already having a splice-site mutation in the other *NtMLO* gene. If this hypothesis is correct, then there should be varieties containing a single mutant of *NtMLO1* or *NtMLO2* among Japanese domestic tobacco cultivars. However, it is unclear when and how these two natural mutations formed, and existence of cultivars harboring a single of the *NtMLO* gene mutation has not been confirmed.

To investigate these hypotheses, we determined the presence of *NtMLO1/2* splice-site mutations in 84 Japanese domestic tobacco cultivars and 94 foreign tobacco varieties using an allele-specific PCR method (Komatsu *et al.* 2020). We identified 14 Japanese domestic cultivars with *NtMLO1/2* splice-site mutations, and we also identified some Japanese domestic cultivars and foreign varieties containing only the *NtMLO1* splice-site mutation. Consequently, we developed a hypothesis for the origins of the two splice-site mutations.

## Materials and Methods

### Materials

Tobacco seeds were obtained from the genetic resources of the Leaf Tobacco Research Center, Japan Tobacco Inc. In total, 84 Japanese domestic cultivars from various tobacco production areas were used for the analysis. In addition, 30 native tobacco varieties of Asia (Philippines, Indonesia, Thailand, India and Nepal), 6 native tobacco varieties of the Americas (Brazil, Colombia and Canada), 35 Oriental tobacco varieties (Turkey, Greece and the former Yugoslavia) and 23 cigar tobacco varieties from various countries (Canada, USA, Colombia, Puerto Rico and the Philippines) were used for the analysis. Many of these varieties have been selected in their respective regions and cultivated for many years. The origin and leaf shape of each variety were provided in the Leaf Tobacco Research Center genetic resources database.

### Splice-site mutation analysis

Genomic DNA was isolated from young leaf tissues using a Gentra Puregene Cell kit (QIAGEN) according to the manufacturer’s instructions. The splice-site mutations in the *NtMLO1/2* genes were analyzed using the duplex allele-specific PCR method described in Komatsu *et al.* (2020). The obtained PCR fragments were separated using a QIAxcel Advanced System (QIAGEN), and the presence of splice-site mutations was determined by the amplification or non-amplification of DNA fragments with the primer pairs specific for the mutant or wild type of each *NtMLO* gene.

### Phylogenetic analysis

Genotyping data for each Japanese domestic cultivar and modern tobacco varieties K326 (flue-cured variety) and TN90 (burley tobacco) as an outgroup were obtained from 30 simple sequence repeat (SSR) markers and 31 loci. The sequences of SSR markers used are provided in Table 1. Each forward primer was labeled with a FAM, VIC, TET or NED fluorescent dye (Applied Biosystems) at the 5ʹ end, and a tail sequence (5ʹ-GTGTCTT-3ʹ) was added to each reverse primer. PCR amplification was performed using a QIAGEN Multiplex kit, modified to three set for multiplex reactions in accordance with Komatsu *et al.* (2020). The PCR products were diluted 50-fold, and 1 μl of Hi-Di formamide (Thermo Fisher Scientific) was added to 10 μl of diluted solution. The mixture was heated for 5 min at 96°C, cooled rapidly on ice, electrophoresed using a 3730xl DNA Analyzer (Life Technology, Applied Biosystems) and analyzed using GeneMapper ver. 4.0 software (Applied Biosystems). A dendrogram was constructed by the neighbor-joining method (Nei *et al.* 1983) using Populations 1.2.3 (Langella 1999), and a phylogenetic tree was constructed using MEGA X software (megasoftware.net). The reliability of each node was evaluated by 1000 trials of the bootstrap method.

### Powdery mildew susceptibility analysis

The susceptibility of tobacco plants to powdery mildew was confirmed using the method described in Fujimura *et al.* (2016). The tobacco plants were grown in clay pots in a greenhouse maintained at 25°C. The powdery mildew spore solution (approximately 2.5 × 10^4^ spore/ml) was splayed on the tobacco plants, and they were checked for disease symptoms at 3 weeks after inoculation.

## Results

### *NtMLO1/2* splice-site mutations in Japanese domestic cultivars

To determine the presence of *NtMLO1/2* splice-site mutations, 84 Japanese domestic cultivars originating from various tobacco production areas were analyzed (Table 2). Among them, 14 cultivars had splice-site mutations in both *NtMLO1* and *NtMLO2* genes and showed high resistance against powdery mildew. Interestingly, 13 of 14 cultivars originated from Kagoshima and the leaf shape of each cultivar is ‘Kokubu’ type. The remaining cultivar, ‘Bingo (Ito)’, was cultivated in Hiroshima and has the ‘Suifu’-type leaf shape.

**Fig. 1.**
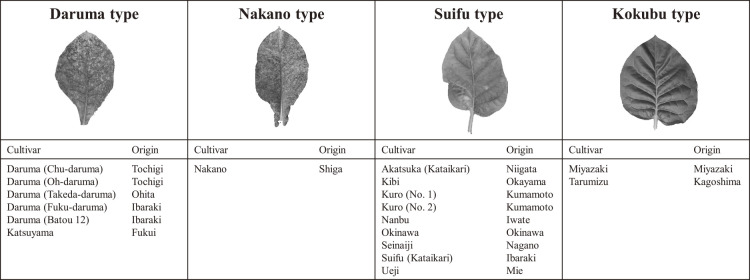
Leaf types of Japanese domestic tobacco cultivars harboring only the *NtMLO1* splice-site mutation. Each cultivar was selected and cultivated in the individual tobacco production area (‘Origin’) of Japan. ‘Daruma’- and ‘Nakano’-type leaves are sessile, and ‘Suifu’- and ‘Kokubu’-type leaves have petioles. All these cultivars are susceptible to powdery mildew. The leaves in the upper part of the figure show the typical shape of each leaf type.

In total, 18 cultivars had a mutation only in the *NtMLO1* splice site. These varieties were from various tobacco production areas, ranging from Iwate to Okinawa, and had four types of leaf shape: ‘Kokubu’ and ‘Suifu’ types, having petioles, and sessile ‘Daruma’ and ‘Nakano’ types (Fig. 1). However, there was no correlation between the *NtMLO1* splice-site mutation and leaf shape, and the cultivars with the ‘Suifu’- or ‘Daruma’-type leaf shape did not necessarily have the *NtMLO1* splice-site mutation. Cultivar ‘Tarumizu (Ishiodori)’ contained splice-site mutations in both *NtMLO1* and *NtMLO2* genes, whereas cultivar ‘Tarumizu’ had a splice-site mutation only in the *NtMLO1* gene, and both had the ‘Kokubu’-type leaf shape. All five ‘Daruma’ group cultivars we tested in this study had *NtMLO1* splice-site mutation. We tested three ‘Suifu’ group cultivars, and only cultivar ‘Suifu (Kataikari)’ had *NtMLO1* splice-site mutation. Here, cultivars with the *NtMLO1* splice-site mutation showed susceptibility against powdery mildew (data not shown). We did not find a variety that has a mutation only in the *NtMLO2* splice site among the tested Japanese domestic cultivars.

### Phylogenetic analysis of the Japanese domestic cultivars

**Fig. 2.**
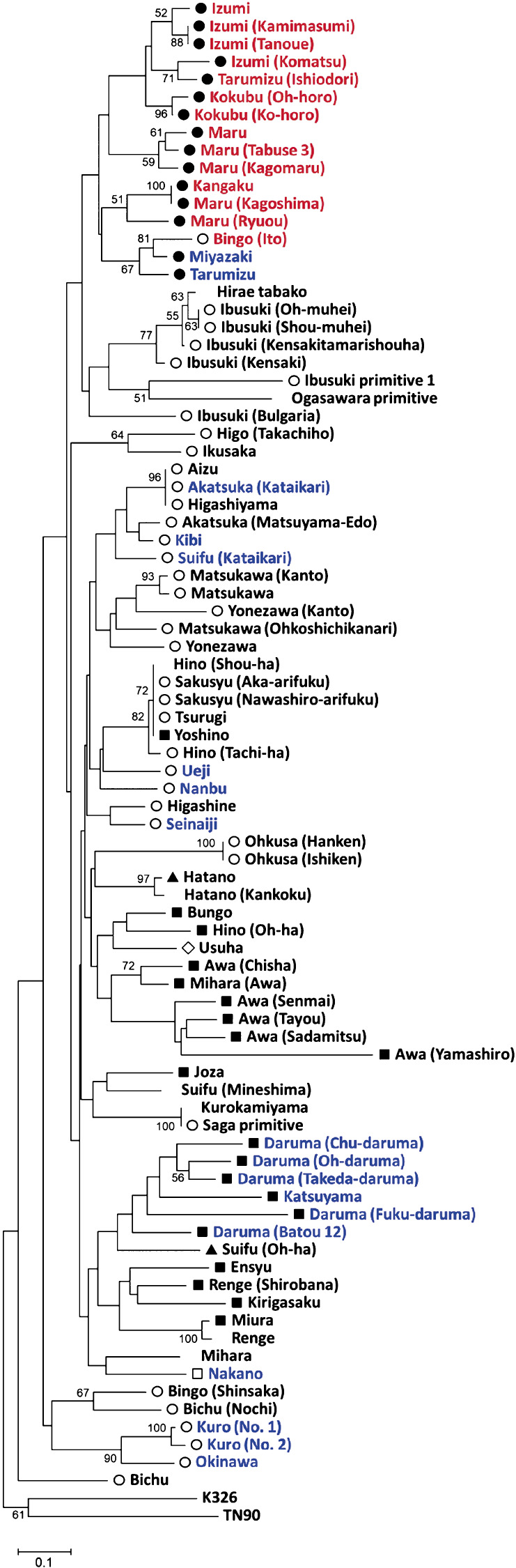
Phylogenetic tree of 84 Japanese domestic tobacco cultivars based on 30 markers. Cultivars in red and blue indicate the *NtMLO1/2* double and *NtMLO1* single mutants, respectively. The symbol at the head of the cultivar name indicates the type of leaf shape; ●: ‘Kokubu’ type, ○: ‘Suifu’ type, ■: ‘Daruma’ type, □: ‘Nakano’ type, ▲: ‘Hatano’ type, and ◇: ‘Usuha’ type. Some cultivars do not have their leaf types listed in the Leaf Tobacco Research Center genetic resource database. The numbers in phylogenetic tree indicate the exceeded bootstrap value by 50. The scale bar at the bottom indicates the genetic distance. Modern tobacco varieties K326 (flue-cured variety) and TN90 (burley tobacco) were added as an outgroup.

We generated a phylogenetic tree of 84 Japanese domestic cultivars (Fig. 2). The cultivars with the *NtMLO1/2* splice-site mutations, including ‘Kokubu’ and ‘Bingo (Ito)’, formed one group. Interestingly, this group contained cultivars ‘Tarumizu’ and ‘Miyazaki’, which have only the *NtMLO1* splice-site mutation, but the ‘Kokubu’-type leaf shape. The cultivars with ‘Suifu’- and ‘Daruma’-type leaf shapes were roughly divided into two major groups within the phylogenetic tree. The cultivars having the *NtMLO1* splice-site mutation and the ‘Suifu’-type leaf shape were distributed throughout the tree branches. However, the cultivars having the *NtMLO1* splice-site mutation and the ‘Daruma’-type leaf shape, which were five ‘Daruma’ group cultivars and cultivar ‘Katsuyama’, formed one group.

### *NtMLO1/2* splice-site mutations in foreign varieties

Most of the cultivars harboring mutations in the splice sites of both *NtMLO1* and *NtMLO2* were cultivated in Kagoshima. The cultivars that harbored only the *NtMLO1* splice-site mutation were from various tobacco production areas of Japan, and no variety having only the *NtMLO2* splice-site mutation was identified among the Japanese domestic tobacco cultivars. Thus, we speculated that the splice-site mutation in the *NtMLO1* gene occurred during the early period of tobacco seed distribution in Japan or before the introduction of tobacco seeds into Japan. Thus, varieties harboring the *NtMLO2* splice-site mutation might be identified by surveying foreign varieties. To confirm this hypothesis, we assessed the presence of *NtMLO1/2* splice-site mutations in various foreign tobacco varieties.

We selected native tobacco varieties derived from various countries, as well as Oriental and cigar tobacco varieties. These varieties had been cultivated in their local areas for long time periods. Because Japan was closed to foreigners from the 17^th^ to 19^th^ centuries, it is unlikely that the tobacco seeds were exported from Japan to foreign countries during this period.

In our analysis, we did not find a foreign tobacco variety with splice-site mutations in both the *NtMLO1* and *NtMLO2* genes. However, the splice-site mutation in the *NtMLO1* gene, which was the same as that in cultivar ‘Kokubu’, was detected in some of the Asian native tobacco varieties of the Philippines, Thailand, India and Nepal, Oriental tobacco varieties of Turkey and the former Yugoslavia, and cigar tobacco varieties of the Philippines, and Florida301 that was bred in USA (Table 3). These results suggest that the *NtMLO1* splice-site mutation is not restricted to Japanese domestic cultivars. The *NtMLO2* splice-site mutation was not found in any tested foreign tobacco variety.

## Discussion

We surveyed the splice-site mutations of the *NtMLO1* and *NtMLO2* genes in various Japanese domestic tobacco cultivars and found that 14 cultivars harbor *NtMLO1/2* splice-site mutations. They formed a distinct group in the phylogenetic tree. Among them, 13 cultivars had the ‘Kokubu’-type leaf shape and originated from Kagoshima. However, tobacco cultivars containing only the *NtMLO1* splice-site mutation were observed from various tobacco production areas of Japan, and their leaf shapes varied. Additionally, tobacco cultivar containing only the *NtMLO2* splice-site mutation was not found. Consequently, we speculated that powdery mildew-resistant tobacco cultivar ‘Kokubu’ was generated relatively recently in the Kokubu area by the occurrence of a spontaneous *NtMLO2* splice mutation the ‘Kokubu’-type ancestral tobacco already harboring the *NtMLO1* splice-site mutation. Then, this *NtMLO1/2* double-mutant tobacco spread to several production areas in Kagoshima and was established as a local domestic cultivar. This may have led to the large number of cultivars with the *NtMLO1/2* splice-site mutations in Kagoshima. Cultivar ‘Bingo (Ito)’ has the *NtMLO1/2* splice-site mutations, but the leaf shape is ‘Suifu’ type and it originates from Hiroshima. The phylogenetic analysis included cultivar ‘Bingo (Ito)’ in the same group as the other cultivars with the *NtMLO1/2* splice-site mutations. Cultivar ‘Bingo (Ito)’ was isolated in 1901 (Ohashi 1984). Therefore, this cultivar is a new variety compared with other ‘Kokubu’ type *NtMLO1/2* double-mutant cultivars, and this cultivar might have been generated by breeding in Hiroshima using the *NtMLO1/2* double-mutant tobacco introduced from Kagoshima.

Cultivars ‘Tarumizu’ and ‘Miyazaki’, which originated in Kagoshima and Miyazaki, respectively, only have a mutation in the *NtMLO1* splice site and have the ‘Kokubu’-type leaf shape. They belong to the group on the phylogenetic tree that contained the *NtMLO1/2* double-mutant cultivars although the bootstrap values are lower than 50 in Fig. 2. Consequently, we speculate that both cultivars may have originated from the *NtMLO1* single-mutant tobacco that is the common ancestor of the *NtMLO1/2* double-mutant cultivars or from progeny of the *NtMLO1/2* double-mutant tobacco in which the *NtMLO2* splice-site mutation was lost.

Our survey of foreign tobacco varieties showed that the *NtMLO1* splice-site mutation occurred not only in Japanese domestic cultivars but also in some of Asian native, Oriental and cigar tobacco varieties. This result indicates that the *NtMLO1* splice-site mutation did not occur in Japan. It is unclear when and where the *NtMLO1* splice-site mutation occurred during the dissemination of tobacco seeds worldwide, but because it was not detected in the tested native varieties of the Americas, this mutation may have occurred early during the spread of tobacco seeds from the Americas to Asia and Japan via Europe. Japanese domestic tobacco cultivars are thought to have been established from a few ancestral varieties introduced into Japan by the Portuguese and/or Spaniards (Ohashi 1984). Therefore, we speculate that two types of tobacco varieties with and without the *NtMLO1* splice-site mutation were included in the tobacco seeds imported into Japan and that both types of seeds were spread through Japan during the national isolation period.

The splice-site mutation in the *NtMLO2* gene was not found in foreign tobacco varieties. This result emphasizes that the *NtMLO2* splice-site mutation is a spontaneous mutation that occurred in a tobacco plant harboring the *NtMLO1* splice-site mutation in Japan.

At present, the cultivation of Japanese domestic tobacco cultivars is decreasing owing to the reduced demand for Japanese domestic tobacco leaves as a tobacco product material, and most of the tobacco varieties cultivated in Japan now are modern flue-cured and burley tobacco varieties used in cigarettes. However, Japanese domestic tobacco cultivars have diverse agronomic traits suitable for each cultivation area. They are useful materials for plant research owing to their diverse genetic traits and have the potential to be good materials for future tobacco breeding.

## Author Contribution Statement

MA, TT and SS designed this study. MA, TK and SS contributed the splice-site mutation analysis. SS and HU contributed the phylogenetic analysis. MA contributed the powdery mildew susceptibility analysis and drafted the manuscript. All authors read and approved the final manuscript.

## Figures and Tables

**Table 1.** Sequences of SSR primers for phylogenetic analysis

Primer pairs	Primer No.	Fowerd primer Sequence	Fluorescent labels	Reverse primer sequence
Group 1	1	TGAGTACATGTTATCTACAAGATCAGGA	FAM	GTGTCTTAATAAAGAATGCTATTCGGTGATACCAG
	2	TGTCTCGTGAAGCATGAA	VIC	GTGTCTTGGAAATGGAGGATCTCGT
	3	AATTCCTTTCTATCTCTAACTACTACTACA	NED	GTGTCTTCTTTACCTTCCATCTTTTTAATTGTGTATC
	4	CCTTTCTGACTTGCTTACGACTACAAA	NED	GTGTCTTGTCTATCACTGACTTTGGTTCCCCTGAA
	5	TGACCACATGAAGGTGCATT	NED	GTGTCTTGAGCTAGAAATAGGTTGTTTGGG
	6	TTTGGTGAGGTGTTACGATAAAGA	FAM	GTGTCTTTCCACACCAAACATCAACTTT
	7	TTCGGCTACCAGCACTATACC	PET	GTGTCTTTCTTATTTGCTGCAGGAATGC
	8	CCCGGCTGATGATATGTGTA	VIC	GTGTCTTTCACGAGGAAGGAGAATGGT
	9*	TTTGGAATCAATAAGACGACAA	FAM	GTGTCTTTTGACCAGTAGGCTTATCACACA
	10	CTCGAACGAACATGTATACTTCGTGTA	PET	GTGTCTTGATTACGAGTCTGTCTCCTTCTTCTAA
	11	GGGCCTACCTATAGAAATAGATATACTTTAAC	PET	GTGTCTTGTGTCTTTCGTATAGATGAGTAACAGAACTTGTCC
Group 2	12	GTCTACGATCCCATTGCTTTTTAT	FAM	GTGTCTTTGTGATTTACGTAATTGGTTTGCT
	13	CAATACCTAAATTTGAGGAGGTTGT	FAM	GTGTCTTTTAATTTTCAGCTAGAGAGAGAGATGAG
	14	ACCCCTCTCCTAATTAATTTCCAAC	VIC	GTGTCTTCGGATCGTAAATTTTAGGCAAGAAG
	15	TTCACAGGGTGGGAAAATGT	FAM	GTGTCTTACTCCTAAACCTCGCCCAAC
	16	GAGCCACATAACCCATACATCTCTATT	NED	GTGTCTTTTACCTGGGAAAGGAAGCTTGTTGATT
	17	CAAACGCCCACTTAGTCTATCCAAAAA	PET	GTGTCTTGTAGAAAAATCAGGTCGGCACATGAGG
	18	GGATGTGGATGCGGGTAA	VIC	GTGTCTTCAAACGGTGCAGTGTTCTGT
	19	CTTCTTCCTAAGCCGAGGGT	NED	GTGTCTTTTGATGATAGAACGCAACTCG
	20	TGATCACACTTGATAGCCTAAAGAA	NED	GTGTCTTCGCACGACCTATACCCATTT
	21	GGAGAGAGATGATATTTAAGTGGTTTCT	PET	GTGTCTTGTCTCTTTTCTAACCTCTCCAACTATTTTATGCAG
Group 3	22	TCCCAGTGAAGATTAGCTTTCAAGA	PET	GTGTCTTGGCACAAAGTATCAGTTAAAGCAAC
	23	GGATCTTGCCCATAATCTTAATTTCTCA	VIC	GTGTCTTGTTCGCTGGATGTTGCAGAGAATTTTT
	24	GTATGCAGGAATGAAATTACCACAATAG	NED	GTGTCTTGAATATTTATGGCTTGTTTCAGACCAC
	25	GTAAAAATGGGGAGACATCACGAAAAC	FAM	GTGTCTTGAAGAGGGAGTTTCCTTTACTTGAGAT
	26	GTTTGCAGATTGCACAGCTT	VIC	GTGTCTTTGCTGAGATCATTGTGAGGC
	27	AGTTGCAGGATTGTTCGCTT	FAM	GTGTCTTCGACTGCAAGAGTTGGCAAT
	28	ATTCTTAAACACTCCACACAAAAACAAG	VIC	GTGTCTTACTATTAAGTTTTGATGAACCCGTAT
	29	GCACAAACTCGATTCAGAACATGCAAT	NED	GTGTCTTAAATCAGTTAGTTAGACGGTGCTAGACG
	30	AGGTTCAATGGTTGGGAGAAATTAAC	PET	GTGTCTTCATGATGTTGTGGTCTTACTTTGTAATG

* No. 9 marker was detected for two loci. Tail sequence (5ʹ-GTGTCTT-3ʹ) was added to the reverse primer.

**Table 2.** *NtMLO1/2* splice-site mutations in 84 Japanese domestic cultivars

Cultivar	*NtMLO1*	*NtMLO2*	Leaf type	Origin
Aizu	W	W	Suifu	Fukushima
Akatsuka (Kataikari)	M	W	Suifu	Niigata
Akatsuka (Matsuyama-Edo)	W	W	Suifu	Niigata
Awa (Chisha)	W	W	Daruma	Tokushima
Awa (Senmai)	W	W	Daruma	Tokushima
Awa (Tayou)	W	W	Daruma	Tokushima
Awa (Yamashiro)	W	W	Daruma	Tokushima
Awa (Sadamitsu)	W	W	Daruma	Tokushima
Bichu	W	W	Suifu	Okayama
Bichu (Nochi)	W	W	Suifu	Okayama
Bingo (Ito)	M	M	Suifu	Hiroshima
Bingo (Shinsaka)	W	W	Suifu	Hiroshima
Bungo	W	W	Daruma	Ohita
Daruma (Chu-daruma)	M	W	Daruma	Tochigi
Daruma (Oh-daruma)	M	W	Daruma	Tochigi
Daruma (Takeda-daruma)	M	W	Daruma	Ohita
Daruma (Fuku-daruma)	M	W	Daruma	Ibaraki
Daruma (Batou 12)	M	W	Daruma	Ibaraki
Ensyu	W	W	Daruma	Shizuoka
Hatano	W	W	Hatano	Kanagawa
Hatano (Kankou)	W	W		Kanagawa
Higashine	W	W	Suifu	Yamagata
Higashiyama	W	W	Suifu	Iwate
Higo (Takachiho)	W	W	Suifu	Kumamoto
Hino (Oh-ha)	W	W	Daruma	Tottori
Hino (Shou-ha)	W	W		Tottori
Hino (Tachi-ha)	W	W	Suifu	Tottori
Hirae tabako	W	W		Kagoshima
Ibusuki (Kensaki)	W	W	Suifu	Kagoshima
Ibusuki (Kensakitamarishouha)	W	W	Suifu	Kagoshima
Ibusuki (Oh-muhei)	W	W	Suifu	Kagoshima
Ibusuki (Shou-muhei)	W	W	Suifu	Kagoshima
Ibusuki (Bulgaria)	W	W	Suifu	Kagoshima
Ibusuki primitive 1	W	W	Suifu	Kagoshima
Ikusaka	W	W	Suifu	Nagano
Izumi	M	M	Kokubu	Kagoshima
Izumi (Komatsu)	M	M	Kokubu	Kagoshima
Izumi (Kamimasumi)	M	M	Kokubu	Kagoshima
Izumi (Tanoue)	M	M	Kokubu	Kagoshima
Joza	W	W	Daruma	Fukuoka
Kangaku	M	M	Kokubu	Kagoshima
Katsuyama	M	W	Daruma	Fukui
Kirigasaku	W	W	Daruma	Chiba
Kibi	M	W	Suifu	Okayama
Kokubu (Oh-horo)	M	M	Kokubu	Kagoshima
Kokubu (Ko-horo)	M	M	Kokubu	Kagoshima
Kuro (No. 1)	M	W	Suifu	Kumamoto
Kuro (No. 2)	M	W	Suifu	Kumamoto
Kurokamiyama	W	W		
Maru	M	M	Kokubu	Kagoshima
Maru (Kagoshima)	M	M	Kokubu	Kagoshima
Maru (Kagomaru)	M	M	Kokubu	Kagoshima
Maru (Ryuou)	M	M	Kokubu	Kagoshima
Maru (Tabuse 3)	M	M	Kokubu	Kagoshima
Matsukawa	W	W	Suifu	Fukushima
Matsukawa (Kanto)	W	W	Suifu	Fukushima
Matsukawa (Ohkoshichikanari)	W	W	Suifu	Fukushima
Mihara	W	W		Hiroshima
Mihara (Awa)	W	W	Daruma	Hiroshima
Miura	W	W	Daruma	Kanagawa
Miyazaki	M	W	Kokubu	Miyazaki
Nakano	M	W	Nakano	Shiga
Nanbu	M	W	Suifu	Iwate
Ogasawara primitive	W	W		Ogasawara-jima
Ohkusa (Hanken)	W	W	Suifu	Aichi
Ohkusa (Ishiken)	W	W	Suifu	Aichi
Okinawa	M	W	Suifu	Okinawa
Renge	W	W		Fukushima
Renge (shirobana)	W	W	Daruma	Saitama
Saga primitive	W	W	Suifu	Saga
Sakusyu (Aka-arifuku)	W	W	Suifu	Okayama
Sakusyu (Nawashiro-arifuku)	W	W	Suifu	Okayama
Seinaiji	M	W	Suifu	Nagano
Suifu (Kataikari)	M	W	Suifu	Ibaraki
Suifu (Oh-ha)	W	W	Hatano	Ibaraki
Suifu (Mineshima)	W	W		Ibaraki
Tarumizu	M	W	Kokubu	Kagoshima
Tarumizu (Ishiodori)	M	M	Kokubu	Kagoshima
Tsurugi	W	W	Suifu	Ishikawa
Ueji	M	W	Suifu	Mie
Usuha	W	W	Usuha	Niigata
Yoshino	W	W	Daruma	Nara
Yonezawa	W	W	Suifu	Yamagata
Yonezawa (Kanto)	W	W	Suifu	Yamagata

This table shows the existence of splice-site mutations in *NtMLO1/2* genes (W: wild type, M: mutant), leaf type (‘Suifu’, ‘Kokubu’, ‘Daruma’, ‘Nakano’, ‘Hatano’ or ‘Usuha’ type), and original cultivation area (indicated by Prefecture name) of each cultivar. Leaf types and origins of some cultivars are unknown (vacant cells).

**Table 3.** *NtMLO1/2* splice-site mutations in 94 foreign tobacco varieties

Variety	*NtMLO1*	*NtMLO2*	Class	Origin
Dork Dang	W	W	Native	Thailand
Hu Chang	W	W	Native	Thailand
Hu Lahm	W	W	Native	Thailand
Slee	M	W	Native	Thailand
Sukhothai	M	W	Native	Thailand
Bansud	M	W	Native	Philippines
Romero	M	W	Native	Philippines
Sinai	W	W	Native	Philippines
Orinoco	W	W	Native	Indonesia
Done Vittanam (Nallapati)	M	W	Native	India
Karravittanam	W	W	Native	India
Medarametlanuta	M	W	Native	India
Naru (Nellore)	W	W	Native	India
Rayala	M	W	Native	India
Toka-Aku	W	W	Native	India
Rayala (No. 1)	M	W	Native	India
RPK	W	W	Native	India
Sazi	W	W	Native	India
Shah-Kot	W	W	Native	India
Sivapuri	W	W	Native	India
Snuff (Eluru)	W	W	Native	India
Thakeri Ramperr	W	W	Native	India
Thatayan	W	W	Native	India
Tholan	W	W	Native	India
Vadamugam	M	W	Native	India
Vazhai Kappal	W	W	Native	India
Nepal 1288	W	W	Native	Nepal
Nepal 8039	M	W	Native	Nepal
Nepal 6184 (Damre Kacho)	M	W	Native	Nepal
Nepal KY	M	W	Native	Nepal
Galpao	W	W	Native	Brasil
Brazilian domestic variety	W	W	Native	Brasil
Carotte	W	W	Native	Brasil
Garcia	W	W	Native	Colombia
Ambalema	W	W	Native	Colombia
Petit Havana	W	W	Native	Canada
Agrinion Djebel	W	W	Oriental	Greece
Agrinion Myrodata	W	W	Oriental	Greece
Agrinion Smyrna Seed	W	W	Oriental	Greece
Basma	W	W	Oriental	
Basma Boukia Paranestion	W	W	Oriental	Greece
Basma Drama Drama	W	W	Oriental	Greece
Basma Drama Ferai	W	W	Oriental	Greece
Basma Kavala Amisiana	W	W	Oriental	Greece
Basma (5A) Kilikis Lakhanas	W	W	Oriental	Greece
Basma Komotini-1 Miskon	W	W	Oriental	Greece
Basma Komotini Ova Kallisti	W	W	Oriental	Greece
Basma Xanthi Ova Bafika	W	W	Oriental	Greece
Basma II Zichna Mesorachi	W	W	Oriental	Greece
Kabakoulak Gumenitsa Gumenitsa	W	W	Oriental	Greece
Kawalla	W	W	Oriental	Greece
Kilikis	W	W	Oriental	Greece
Kozani	W	W	Oriental	Greece
Mahala	W	W	Oriental	Greece
Nigrita	W	W	Oriental	Greece
Sochos 1	W	W	Oriental	Greece
Dzebel	W	W	Oriental	Ex-Yugoslavia
Otlija	M	W	Oriental	Ex-Yugoslavia
Prilep	W	W	Oriental	Ex-Yugoslavia
Ravnijak	M	W	Oriental	Ex-Yugoslavia
Yaka Bolsunov	W	W	Oriental	Ex-Yugoslavia
Akhissar-1	W	W	Oriental	Turkey
Akhissar-1	W	W	Oriental	Turkey
Bafra (Black sea)	M	W	Oriental	Turkey
Bursa	M	W	Oriental	Turkey
Izmir	W	W	Oriental	Turkey
Samsun	M	W	Oriental	Turkey
Samsun Holmes	M	W	Oriental	Turkey
Smyrna	W	W	Oriental	Turkey
Welwendo	W	W	Oriental	
Zihina	W	W	Oriental	
Beinhart1000-1	W	W	Cigar	USA
Connecticut	W	W	Cigar	USA
Florida301	M	W	Cigar	USA
Havana	W	W	Cigar	USA
Warne	W	W	Cigar	USA
Ottawa705	W	W	Cigar	Canada
Cubita	W	W	Cigar	Colombia
Olor	W	W	Cigar	Puerto Rico
Espado	M	W	Cigar	Philippines
Manilla	M	W	Cigar	Philippines
Manilla (Cagayan)	M	W	Cigar	Philippines
Oxsinisirn	M	W	Cigar	Philippines
Oxviz	M	W	Cigar	Philippines
Pampano	M	W	Cigar	Philippines
Repollo	M	W	Cigar	Philippines
Simmaba	M	W	Cigar	Philippines
Simox	W	W	Cigar	Philippines
Vizcaya	M	W	Cigar	Philippines
Vizoxviz	M	W	Cigar	Philippines
Java	M	W	Cigar	
Penn Leaf1	W	W	Cigar	
Sumatra	W	W	Cigar	
Tuta	W	W	Cigar	

This table shows the existence of splice-site mutations in *NtMLO1/2* genes (W: wild type, M: mutant), as well as the class (Native: native variety, Oriental: oriental tobacco variety and Cigar: cigar tobacco variety) and origin (indicated by country name) of the varieties examined. The origins of some varieties are not listed in the Leaf Tobacco Research Center genetic resource database. Ex-Yugoslavia: the former Yugoslavia.
